# Supplementary material for: Artificial Intelligence in Renal Cell Carcinoma Histopathology: Current Applications and Future Perspectives
Source: Diagnostics (Basel). 2023 Jul 6;13(13):2294. doi: 10.3390/diagnostics13132294 (PMC10340141; doi:10.3390/diagnostics13132294)
Supplement: Supplementary file 1 [file diagnostics-13-02294-s001.zip › diagnostics-2454617-supplementary.pdf]

Supplementary material

1. Search strategy

Search strategy for the MEDLINE database

Search:

((((("Kidney Neoplasm*"[Mesh] OR "Renal Cell Carcinoma"[tiab] OR "Renal Cancer*"[tiab]) AND ("Artificial Intelligence"[Mesh] OR "deep learning"[tiab] OR "machine learning"[tiab]) AND ("Pathomics"[tiab] OR "Patholog*"[tiab] OR "Histolog*"[tiab] OR "Histopatholog*"[tiab] OR "whole slide*"[tiab])) NOT (Tomograph*)) NOT (MRI)) NOT (CT-based)) NOT (Radiomic)

2. Analyzed models datasets

Table S1. AI models datasets for diagnosis and subtyping

METHOD	Test cohort dataset	External validation dataset
Fenstermaker et al.	TGCA*	N.A.
Zhu et al.	Dartmouth-Hitchcock Medical Center, New Hampshire, USA	TGCA
Chen et al.	TGCA	Shanghai General Hospital
Tabibu et al.	TGCA	N.A.
Abdeltawab et al.	University of Louisville, Louisville, KY, USA	NIH

Table S2. AI models datasets for grading

METHOD	Test cohort dataset	External validation dataset
Yeh et al.	University of Pittsburgh Medical Center	N.A.
Holdbrook et al.	Singapore General Hospital	N.A.
Tian et al.	TGCA	N.A.

Table S3.AI methods datasets for prognostic models

METHOD	Test cohort dataset	External validation dataset
Ning et al.	TGCA	N.A.
Cheng et al.	TCGA	N.A.
Schulz et al.	TGCA	University Medical Center of Mainz, Germany

Table S4. AI models datasets for molecular morphologic connection and therapy response predictions

METHOD	Test cohort dataset	External validation dataset
Marostica et al.	TGCA	1) NCI Clinical Proteomic Tumor Analysis Consortium (CPTAC)<br>2) Brigham and Women's Hospital Department of Pathology (Boston, MA)
Zhu et al.	Dartmouth-Hitchcock Medical Center, New Hampshire, USA	TGCA
Go et al.	Asan Medical Center, Seoul, South Korea	N.A.
Zheng et al.	GDC data portal, National Cancer Institute	N.A.
